# Supplementary material for: Testing for treatment effect twice using internal and external controls in clinical trials
Source: J Causal Inference. Author manuscript; Available in PMC 2026 Jun 27. (PMC13309237; doi:10.1515/jci-2022-0018)
Supplement: Supplementary Material [file NIHMS2185723-supplement-Supplementary_Material.pdf]

Supplementary material

S1 Supplementary materials

S2 Additional type I error and power calculations

We conduct the type I error calculation in two approaches. For T_1, $T_{2,\Delta_0}(w)$ and the combined test $T_{c,\Delta_0}(w)$, the theoretical version is calculated using formulas in (1), (2), and (5), respectively; the empirical version is obtained from 10,000 simulation repetitions. For comparison, we also include a naive combined test $\widetilde{T}_{c,\Delta_0}(w)$ that performs both T_1 and $T_{2,\Delta_0}(w)$ without adjusting for multiple testing, i.e., it rejects H_0 if $\max(T_1, T_{2,\Delta_0}(w)) \geq z_{1-\alpha}$. In each repetition, n_1 treated subjects are generated from $Y^{(1)}|D = 1\sim N(0, \sigma_1^2)$, n_0 internal controls from $Y^{(0)}|D = 1\sim N(-\theta^\star, \sigma_0^2)$, and n_e external controls from $Y^{(0)}|D = 0\sim N(-\theta^\star - \Delta^\star, \sigma_e^2)$. The results are in Table S1. From Table S1, we see that the type I error rates of T_1, $T_2(1/4)$, $T_c(1/4)$ are close to or below the significance level. However, using the naive combined test $\widetilde{T}_{c,\Delta_0}(w)$ can lead to inflated type I error.

The power calculation is performed similarly; the only difference is that the empirical power is obtained from 3,000 simulation repetitions. The results are in Table S2.

Table S1: Empirical and theoretical type I error (in %) for T_1, $T_{2,\Delta_0}(w)$, the combined test $T_{c,\Delta_0}(w)$, and the naive combined test $\widetilde{T}_{c,\Delta_0}(w)$ with $w = 1/4$, where $\theta_0 = 0$, $\Delta^\star = 0.2$, $n_1 : n_0 : n_e = 2 : 1 : 3$, $\sigma_1 = \sigma_0 = \sigma_e = 1$, and $\alpha = 2.5\%$

		Empirical type I error				Theoretical type I error			
Δ_0	n_1	T_1	$T_2(1/4)$	$T_c(1/4)$	$\widetilde{T}_c(1/4)$	T_1	$T_2(1/4)$	$T_c(1/4)$	$\widetilde{T}_c(1/4)$
0.2	50	2.9	2.6	2.8	4.6	2.5	2.5	2.5	4.2
	100	2.7	2.4	2.7	4.4	2.5	2.5	2.5	4.2
	150	2.6	2.9	2.7	4.6	2.5	2.5	2.5	4.2
	200	2.6	2.7	2.7	4.3	2.5	2.5	2.5	4.2
0.3	50	2.9	0.9	2.0	3.4	2.5	0.8	1.7	2.9
	100	2.7	0.5	1.7	3.0	2.5	0.5	1.6	2.7
	150	2.6	0.4	1.7	2.8	2.5	0.3	1.5	2.6
	200	2.6	0.2	1.6	2.7	2.5	0.2	1.5	2.6
0.4	50	2.9	0.3	1.7	3.0	2.5	0.2	1.5	2.6
	100	2.7	0.1	1.6	2.7	2.5	0.1	1.5	2.5
	150	2.6	0.0	1.6	2.6	2.5	0.1	1.5	2.5
	200	2.6	0.0	1.6	2.6	2.5	0.1	1.5	2.5
0.6	50	2.9	0.0	1.7	2.9	2.5	0.1	1.5	2.5
	100	2.7	0.0	1.6	2.7	2.5	0.1	1.5	2.5
	150	2.6	0.0	1.6	2.6	2.5	0.1	1.5	2.5
	200	2.6	0.0	1.6	2.6	2.5	0.1	1.5	2.5

The empirical version is based on 10,000 repetitions. In the table, we omit the Δ_0 subscript for notational simplicity.

Table S2: Empirical power (in %, based on 3,000 repetitions) for T_1, $T_{2,\Delta_0}(w)$ and the combined test $T_{c,\Delta_0}(w)$ with $w = 1/4$ or w_{opt}, where $\theta_0 = 0$, $\Delta^* = 0.2$, $n_1 : n_0 : n_e = 2 : 1 : 3$, $\sigma_1 = \sigma_0 = \sigma_e = 1$, and $\alpha = 2.5\%$

		$\theta^* = 0.2$					$\theta^* = 0.3$					$\theta^* = 0.4$				
Δ_0	n_1	T_1	$T_2(1/4)$	$T_2(w_{\text{opt}})$	$T_c(1/4)$	$T_c(w_{\text{opt}})$	T_1	$T_2(1/4)$	$T_2(w_{\text{opt}})$	$T_c(1/4)$	$T_c(w_{\text{opt}})$	T_1	$T_2(1/4)$	$T_2(w_{\text{opt}})$	$T_c(1/4)$	$T_c(w_{\text{opt}})$
0.2	50	13.5	21.7	22.3	20.6	20.6	24.2	42.4	42.4	38.2	38.2	38.2	64.3	64.7	58.9	58.9
	100	21.6	36.0	36.0	32.8	32.8	39.6	68.9	68.9	64.2	64.2	62.6	89.8	89.7	87.2	87.1
	150	29.3	50.6	50.9	46.5	46.5	57.3	84.7	84.7	80.4	80.4	80.7	97.9	97.9	97.0	97.0
	200	37.7	64.2	64.2	58.9	58.7	69.2	93.8	93.8	91.8	91.8	90.9	99.6	99.6	99.3	99.3
0.3	50	13.5	12.3	14.5	13.8	14.1	24.2	26.6	30.3	28.0	28.6	38.2	48.2	50.9	47.0	47.2
	100	21.6	17.1	22.1	21.1	22.2	39.6	45.2	48.8	46.0	46.1	62.6	75.1	76.7	73.7	74.2
	150	29.3	24.0	30.3	28.6	30.4	57.3	60.5	65.9	62.7	63.7	80.7	90.6	92.1	89.6	90.0
	200	37.7	30.5	39.9	37.4	38.9	69.2	75.0	78.3	76.2	76.7	90.9	96.7	97.5	96.8	97.1
0.4	50	13.5	5.6	13.5	10.4	13.5	24.2	15.7	24.3	22.5	24.4	38.2	30.8	40.7	38.3	40.3
	100	21.6	6.3	21.6	16.8	21.6	39.6	22.7	39.7	36.7	39.8	62.6	53.6	65.1	62.7	64.6
	150	29.3	7.5	29.3	23.5	29.3	57.3	32.4	57.3	51.1	57.5	80.7	70.0	83.1	79.9	82.5
	200	37.7	9.1	37.7	30.8	37.7	69.2	41.4	69.2	64.6	69.3	90.9	83.7	92.9	91.5	92.4
0.6	50	13.5	0.9	13.5	9.2	13.5	24.2	3.0	23.7	18.7	24.2	38.2	9.5	38.2	31.7	38.2
	100	21.6	0.1	21.6	15.8	21.6	39.6	2.5	39.1	32.6	39.7	62.6	12.3	62.6	54.2	62.6
	150	29.3	0.1	29.3	22.4	29.3	57.3	2.5	56.4	47.5	57.3	80.7	17.2	80.7	74.3	80.7
	200	37.7	0.1	37.7	30.2	37.7	69.2	2.6	68.5	61.3	69.2	90.9	21.4	90.9	86.5	90.9

We omit the Δ_0 subscript for notational simplicity.

S3 Proof of (3)

To maximize asymptotic power of $T_{2,\Delta_0}(w)$ in (2), we find the w that minimizes

$$g(w) = \frac{(\theta_0 - \theta^*) + (1 - w)(\Delta_0 - \Delta^*)}{\sqrt{\pi_1^{-1}\sigma_1^2 + w^2\pi_0^{-1}\sigma_0^2 + (1 - w)^2 n_r n_e^{-1}\sigma_e^2}}.$$

To simplify notations, let $a = (\theta_0 - \theta^*), b = (\Delta_0 - \Delta^*), c = \pi_1^{-1}\sigma_1^2, d = \pi_0^{-1}\sigma_0^2, e = n_r n_e^{-1}\sigma_e^2$. When $w = 1$, the $g(w) < 0$ as $\theta^* - \theta_0 > 0$, thus the w should satisfy both $a + b(1 - w) < 0$ and maximize

$$f(w) \coloneqq g(w)^2 = \frac{(a + b(1 - w))^2}{c + dw^2 + e(1 - w)^2}.$$

The derivative of $f(w)$ is

$$\begin{aligned} f'(w) &= \frac{2(a + b(1 - w))(-b)(c + dw^2 + e(1 - w))^2 - (2dw + 2e(w - 1))(a + b(1 - w))^2}{(c + dw^2 + e(1 - w)^2)^2} \\ &= \frac{-2(a + b(1 - w))((bc - ae) + (ad + ae + bd)w)}{(c + dw^2 + e(1 - w)^2)^2}. \end{aligned}$$

The maxima $w \in [0, 1]$ could only be among $w = \frac{ae - bc}{ad + ae + bd}$, $w = 0$, or $w = 1$.

Since $a < 0, b \geq 0, c, d, e > 0$:

(1) when $ad + ae + bd > 0$, that is, $-b/a = \frac{\Delta_0 - \Delta^*}{\theta^* - \theta_0} > (d + e)/d$, we have $w = \frac{ae - bc}{ad + ae + bd} < 0$, so it cannot be a maxima. Also recall that we need to maintain $a + b(1 - w) < 0$ at the maxima. However when $w = 0$, $a + b(1 - w) = a + b > 0$. Thus, the maxima is $w = 1$ in this case.

(2) when $ad + ae + bd < 0$, that is, $-b/a = \frac{\Delta_0 - \Delta^*}{\theta^* - \theta_0} < (d + e)/d$. In this case, $f'(1) = \frac{-2a(bc + ad + bd)}{(c + d)^2}$ and $f'(0) = \frac{-2(a + b)(bc - ae)}{(c + e)^2}$. Further consider:

(2.1) If $-b/a < \frac{d}{c + d}$, i.e., $bc + ad + bd < 0$, then $f'(1) = \frac{-2a(bc + ad + bd)}{(c + d)^2} < 0$ concludes $w = 1$ cannot be a maxima. Also in this case $a + b < 0$, so $f'(0) = \frac{-2(a + b)(bc - ae)}{(c + e)^2} > 0$ concludes $w = 0$ cannot be a maxima. In this case, the maxima is $w = \frac{ae - bc}{ad + ae + bd}$.

(2.2) If $-b/a > \frac{d}{c + d}$, then for $w = \frac{ae - bc}{ad + ae + bd}$ is not in $[0, 1]$ as $1 - w = \frac{ad + bd + bc}{ad + ae + bd} < 0$. In this case, the maxima would only be $w = 0$ or $w = 1$. Further assume:

(2.2.1) If $-b/a > 1$, then $g(0) > 0, g(1) < 0$, then $w = 1$ is the maxima.

(2.2.2) if $\frac{d}{c + d} < -b/a < 1$, $g(0) < 0, g(1) < 0$. $f(0) = \frac{(a + b)^2}{c + e}$, $f(1) = \frac{a^2}{c + d}$. Thus, we have $f(0)/f(1) = \left(\frac{a + b}{a}\right)^2 \frac{c + d}{c + e} < \left(\frac{a + b}{a}\right)^2 \frac{c + d}{c} < \frac{c}{c + d} < 1$. The second last inequation holds as $(\frac{-c}{c + d}) < -(a + b)/a < 0$. In this case, the maxima is $w = 1$.

In summary,

(1) When $-b/a > \frac{d}{c + d}$, the maximum is achieved at $w = 1$.

(2) When $-b/a < \frac{d}{c + d}$, the maximum is achieved at $w = \frac{ae - bc}{ad + ae + bd}$.
